# Surface Modification Using Polydopamine-Coated Liquid Metal Nanocapsules for Improving Performance of Graphene Paper-Based Thermal Interface Materials

**DOI:** 10.3390/nano11051236

**Published:** 2021-05-07

**Authors:** Jingyao Gao, Qingwei Yan, Xue Tan, Le Lv, Jufeng Ying, Xiaoxuan Zhang, Minghui Yang, Shiyu Du, Qiuping Wei, Chen Xue, He Li, Jinhong Yu, Cheng-Te Lin, Wen Dai, Nan Jiang

**Affiliations:** 1 Key Laboratory of Marine Materials and Related Technologies, Zhejiang Key Laboratory of Marine Materials and Protective Technologies, Ningbo Institute of Materials Technology and Engineering, Chinese Academy of Sciences, Ningbo 315201, China; gaojingyao@nimte.ac.cn (J.G.); yanqingwei@nimte.ac.cn (Q.Y.); tanxue@nimte.ac.cn (X.T.); lvle@nimte.ac.cn (L.L.); yingjunfeng@nimte.ac.cn (J.Y.); zhangxiaoxuan@nimte.ac.cn (X.Z.); xuechen@nimte.ac.cn (C.X.); lihe@nimte.ac.cn (H.L.); yujinhong@nimte.ac.cn (J.Y.); 2 Center of Materials Science and Optoelectronics Engineering, University of Chinese Academy of Sciences, Beijing 100049, China; 3 College of Materials Science and Engineering, Hunan University, Changsha 410082, China; 4 Ningbo Institute of Materials Technology and Engineering, Chinese Academy of Sciences, Ningbo 315201, China; myang@nimte.ac.cn; 5 Engineering Laboratory of Advanced Energy Materials, Ningbo Institute of Materials Technology and Engineering, Chinese Academy of Sciences, Ningbo 315201, China; dushiyu@nimte.ac.cn; 6 School of Materials Science and Engineering, Central South University, Changsha 410083, China; qiupwei@csu.edu.cn

**Keywords:** liquid metal, graphene paper, surface modification, thermal contact resistance, thermal interface materials

## Abstract

Given the thermal management problem aroused by increasing power densities of electronic components in the system, graphene-based papers have raised considerable interest for applications as thermal interface materials (TIMs) to solve interfacial heat transfer issues. Significant research efforts have focused on enhancing the through-plane thermal conductivity of graphene paper; however, for practical thermal management applications, reducing the thermal contact resistance between graphene paper and the mating surface is also a challenge to be addressed. Here, a strategy aimed at reducing the thermal contact resistance between graphene paper and the mating surface to realize enhanced heat dissipation was demonstrated. For this, graphene paper was decorated with polydopamine EGaIn nanocapsules using a facile dip-coating process. In practical TIM application, there was a decrease in the thermal contact resistance between the TIMs and mating surface after decoration (from 46 to 15 K mm^2^ W^−1^), which enabled the decorated paper to realize a 26% enhancement of cooling efficiency compared with the case without decoration. This demonstrated that this method is a promising route to enhance the heat dissipation capacity of graphene-based TIMs for practical electronic cooling applications.

## 1. Introduction

Along with the integration and miniaturization of electronics devices, the thermal management issue caused by the increasing power densities of electronic components is an important area of research [1,2,3,4,5]. Thermal management focuses on the efficient transfer of heat from a hot spot to the surrounding atmosphere. One of the crucial aspects and frequent bottlenecks is increasing the thermal conduction at the interface between the materials [6,7,8]. When two solid surfaces are joined, the actual contact can be as low as 1–2%, which can be attributed to the surface roughness and leads to air (thermal conductivity: 0.026 W m^−1^ K^−1^), filling out the remaining area and results in an obvious temperature drop across the interface [9]. To address this, a thermal interface material (TIM) is commonly applied to replace the void between the two mating surfaces [10,11,12,13]. The thermal performance of TIM is usually evaluated according to its thermal interface resistance (RTIM), which can be expressed by the following formula:

*R_TIM_ = R_Contact_ + BLT/κ_TIM_* (1)

where *R_Contact_* is the thermal contact resistance at the interface between the TIM and the mating surface, *κ_TIM_* is the through-plane thermal conductivity of the TIM, and the *BLT* is the bond line thickness of the applied TIMs under packaging [9]. Thus, in order to meet the ever-increasing heat dissipation requirement, TIMs with high thermal conductivity, low thermal contact resistance, and a relatively low *BLT* are in high demand [14]. Currently, the commercial silicon-based TIMs are prepared by the direct coupling of polymer and thermally conductive fillers (Al_2_O_3_, BN, etc.), having an enhanced thermal conductivity of 1–5 W m^−1^ K^−1^. However, it can be difficult for conventional TIMs to meet the ever-increasing heat dissipation requirement with the continuous improvement of the microelectronics industry.

Graphene, a honeycomb two-dimensional material, possesses good thermal and electrical property [15,16,17]. Thanks to its excellent intrinsic thermal conductivity (3500–5300 W m^−1^ K^−1^) and advantages for mass production [18,19,20], graphene-based paper has attracted significant attention for the development of TIMs [21,22,23,24,25,26,27,28,29]. Feng et al. developed a graphene paper-like TIM with a thickness in the range of 90–120 μm; it demonstrated good heat dissipation for CPU cooling because of its high through-plane thermal conductivity of 9 W m^−1^ K^−1^ [23]. Nan et al. applied nanodiamond particles for intercalation into functionalized graphene oxide to construct a composite graphene paper with adjustable thickness, enhancing the thermal conductivity of graphene paper by 33%, which enabled improving the heat dissipation performance for LED cooling [24]. According to Formula (1), these works focused on enhancing the through-plane thermal conductivity of TIM to decrease the thermal interface resistance of TIM. However, in our previous work [22], we found that when the BLT of TIM is sufficiently low (≤250 μm), the intrinsic thermal resistance of TIM contributed by the thermal conductivity accounts for only a small portion of the total thermal resistance (14 of 61 K mm^2^ W^−1^). This results in the thermal contact resistance dominating the thermal interface resistance of the TIM (47 K mm^2^ W^−1^ of 61 K mm^2^ W^−1^). Hence, reducing the thermal contact resistance between graphene paper and the mating surface is an urgent issue for practical thermal management applications [30].

Liquid metal alloys, for instance, EGaIn (liquid metal alloy composed of Ga and In), have been studied for thermal management because of their high thermal conductivity, low thermal resistance, low viscosity, and low toxicity [31,32,33,34]. Ji et al. decorated liquid metal alloys on the surface of the TIM made of carbon nanotubes, which reduced the thermal interface resistance of the TIM by 26% [35]. However, the pump out effect caused by its low viscosity and poor interface wettability with graphene, which is caused by the high surface tension of liquid metal alloys, limits its direct application for reducing the thermal contact resistance between the graphene-based TIM and the mating surface [32,36,37]. In this work, we decorated the graphene paper surface with polydopamine (PDA)-coated EGaIn nanocapsules, which were fabricated via sonication of bulk EGaIn in an aqueous dopamine hydrochloride solution. The dopamine not only served as a surfactant to stabilize the EGaIn nanodroplet during the sonication process but also immobilized the EGaIn nanocapsules on the graphene paper surface to prevent the EGaIn from flowing. Compared with its undecorated counterpart, the thermal contact resistance of the decorated graphene paper reduced to 33% (from 46 to 15 K mm^2^ W^−1^) under 500 kPa compression. Finally, practical application of the TIM for LED heat dissipation also demonstrated the superior performance of decorated graphene paper for cooling electronic devices.

## 2. Materials and Methods

### 2.1. Materials

Graphene sheets were fabricated from graphite powder (purchased from Ningbo Morsh Technology Co., Ltd., Ningbo, China) via an intercalation and exfoliation method. Characterization of the graphene sheets was carried out in our previous work [38]. EGaIn (composed of 75.5 wt.% Ga and 24.5 wt.% In) with a melting point of 288.9 K was supplied by Sigma-Aldrich. Co., Ltd., St. Louis, MO, USA. Dopamine hydrochloride and ethanol tris were supplied by Sinopharm Chemical Reagent Co., Ltd., Shanghai, China and used without further purification.

### 2.2. Sample Preparation

Step 1: We fabricated PDA-coated EGaIn (PDA-EGaIn) nanocapsules via a facile sonication method, as follows. First, a 1.5 g EGaIn drop was placed in a beaker with 150 mL aqueous dopamine hydrochloride solution (1 mg mL^−1^). Then, a tip sonicator (JY92-IIN, Scientz, Ningbo, China) was applied to fragment the EGaIn droplet in the solution. The sonication time was 60 min with an applied power of 500 W at a temperature of 298 K. After sonication, we added 1.5 mmol tris base into the suspension to provide a weak alkaline environment (pH = 8.5) for the self-polymerization of dopamine. Step 2: We fabricated the graphene paper via a facile filtration process. First, 500 mg of graphene sheets were uniformly dispersed in ethanol via ultrasonication, and then, the dispersion was filtered to obtain the graphene paper using a Teflon filter membrane with the pore size of 0.22 μm. The graphene paper was further decorated by the suspension from step one via a dip-coating method for 6 h, which was followed by sequentially washing with DI water and ethanol.

### 2.3. Characterization

The morphology of the PDA-EGaIn nanocapsules, graphene sheets, and DGP were investigated via scanning electronic microscopy (FE-SEM, Quanta FEG250, FEI, Waltham, USA). The core–shell structure of the PDA-EGaIn nanocapsules was further characterized by Fourier infrared spectroscopy (MB3000, ABB, Zurich, Switzerland) and transmission electron microscopy (F200×, The Thermo Scientific, Waltham, MA USA). High-angle annular dark-field and energy-dispersive X-Ray spectroscopy (EDS) were carried out using the scanning transmission electron microscopy (STEM) mode of the TEM. The thermal conductivity of the graphene paper can be calculated by the equation *λ* = *α* × *C_p_* × *ρ*, in which thermal diffusivity (*α*) was measured using LFA 467 HyperFlash^®^ system (NETZSCH, Selbu, Germany) and specific heat capacity (*C_p_*) was evaluated using a differential scanning calorimeter (DSC) (PYRIS Diamond™, Perkin Elmer, Waltham, USA). An infrared camera (Fluke, Ti400, Everett, WA, USA) was used to capture IR images.

## 3. Results

**Figure 1:**
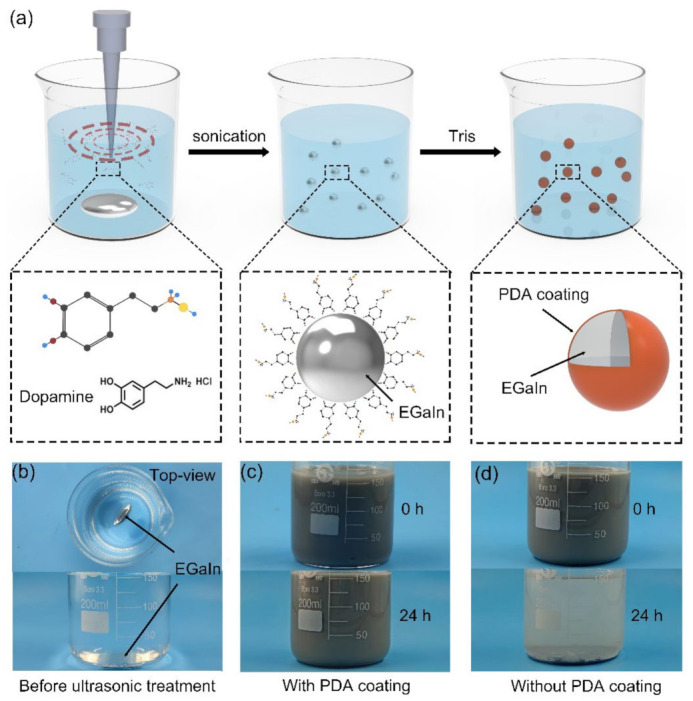
(**a**) Schematic of the fabrication process of the PDA-EGaIn nanocapsules. Images of the EGaIn droplet (**b**) before and after sonication (**c**) with the PDA coating and (**d**) without the PDA coating.

Figure 1a shows the fabrication process for the PDA-EGaIn nanocapsules. During the ultrasonication process, the bulk EGaIn droplet broke into nanodroplets with the help of dopamine hydrochloride in an aqueous solution. The dopamine served as a surfactant to stabilize the EGaIn nanodroplets in water. Since dopamine tends to self-polymerize and then form a thin PDA coating on various surfaces under alkaline conditions (pH > 7.5) without premodification, the weak alkaline environment (PH > 8.5) provided by Tris base facilitated the formation of the PDA coating on the EGaIn nanodroplets [39,40,41,42]. The stabilization effect of the PDA coating on the EGaIn nanodroplet can be directly observed from Figure 1b–d. After 1 h ultrasonication treatment, the bulk EGaIn droplet shown in Figure 1b transformed to the EGaIn ink shown in Figure 1c. After standing for 24 h, the EGaIn ink remained unchanged, which could be attributed to the PDA coating layer serving as a barrier that prevented droplet aggregation [43]. Compared with the suspension of EGaIn nanodroplets sonicated in deionized (DI) water shown in Figure 1d, the higher zeta potential (shown in Figure S1) ensured the stability of the EGaIn ink.

**Figure 2:**
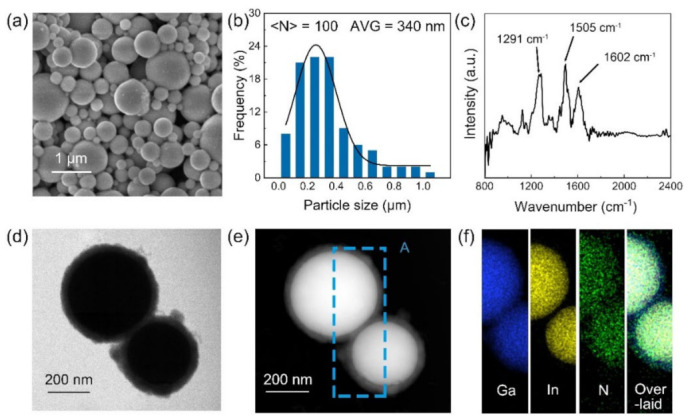
(**a**) SEM image and (**b**) lateral size distribution of the PDA-EGaIn nanocapsules. (**c**) IR spectra of the PDA-EGaIn nanocapsules. (**d**) TEM and (**e**) STEM images of the individual nanocapsule. (**f**) Ga (blue), In (yellow), N (green), and the overlaid elemental mapping of the nanocapsules detected from position A in (**e**).

The morphology of the as-prepared PDA-EGaIn nanocapsules was investigated via scanning electron microscopy (SEM), as shown in Figure 2a. Under the action of the surface tension, the nanodroplets maintained a spherical shape because the ultrasonic treatment temperature (298 K) was higher than the melting point of EGaIn (288.9 K). The nanocapsules particle size distribution could also be observed from the SEM image, as shown in Figure 2b. The diameter of the nanocapsules ranged from 50 nm to 1 μm, with an average size of 340 nm, showing a smaller size compared with EGaIn sonicated in DI water (Figure S2), further proving the anti-aggregation effect of adding dopamine. For characterizing the core–shell structure of the nanodroplet, FT-IR was used to confirm the PDA coating layer on the nanodroplet, the results of which are shown in Figure 2c. The absorption bands at 1602 cm^−1^ (stretching vibration of the aromatic ring and bending vibration of N–H), 1505 cm^−1^ (shearing vibration of N–H), and 1291 cm^−1^ (stretching vibration of phenolic C–O) indicate the presence of PDA on the nanodroplet [44,45]. The core–shell structure of the PDA-EGaIn nanocapsules was further investigated via TEM. A typical core–shell structure TEM image is shown in Figure 2d. It is clear that the rough PDA coating layer encapsulated the smooth EGaIn nanodroplets surface without any voids between the EGaIn/PDA interface. Figure 2e shows the high-angle annular dark-field STEM results, which more clearly demonstrate the core–shell structure, and the average thickness of the PDA coating layer was ≈ 25 nm. EDS elemental mapping was used to characterize the composition of the core–shell structure, as shown in Figure 2f. Ga and In were uniformly distributed only in the core of nanocapsules, while N, which indicates the presence of PDA, was found across the nanocapsules, further confirming the core–shell structure.

**Figure 3:**
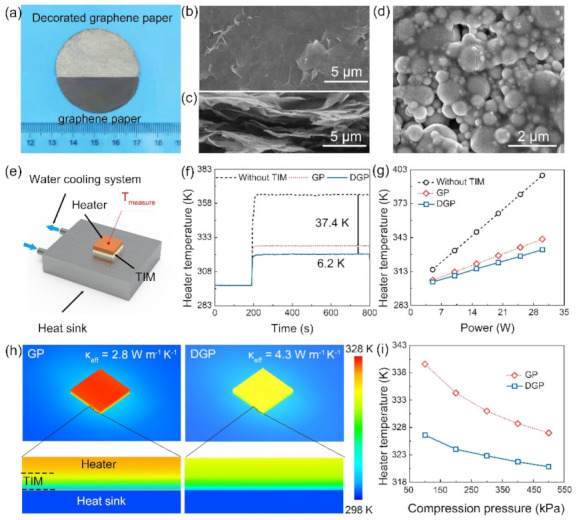
(**a**) Image of decorated graphene paper (top) and pristine graphene paper (bottom). (**b**) Top view and (**c**) cross-sectional SEM images of GP. (**d**) Top view SEM image of DGP. (**e**) Schematic configuration of the TIM performance measurement system and packaging of the applied TIM using vertical compression (500 kPa). (**f**) Temperature evolution of the heater as a function of heating time at a heater power of 20 W. (**g**) Various applied powers after heating for 600 s. (**h**) Comparison of heat dissipation capability based on simulated profiles of GP and DGP. (**i**) Temperature of the heater as a function of the applied compression pressure.

The fabricated PDA-EGaIn nanocapsules were used to decorate the surface of graphene paper TIM via a facile dip-coating method to reduce the thermal contact resistance between the graphene paper and mating surface. Figure 3a shows photos of the graphene paper before and after decoration with the PDA-EGaIn nanocapsules, and they exhibit an obvious difference in color. The differences in the microscopic morphology were investigated via SEM, as shown in Figure 3b and d. Graphene paper prepared via the filtration process exhibited a smooth surface (Figure 3b) and a layer-by-layer stacking structure (Figure 3c). During the decoration process, dopamine self-polymerized and adhered to the graphene paper surface with PDA-EGaIn nanocapsules, as shown in Figure 3d. To investigate the effect of decoration on reducing the thermal contact resistance between graphene paper and the mating surface, a performance measurement system was developed to simulate the practical heat dissipation process in electronic devices, as schematically illustrated in Figure 3e. Graphene paper (GP) and decorated graphene paper (DGP) with equal lateral sizes (1 × 1 cm^2^) and BLT of 250 μm were applied as TIM packaged between the heat source and the heat sink under a packaging pressure of 500 kPa, where heat generated by this measurement system was extracted via the continuous pumping of cooling water through the liquid-cooled Al heat sink to reach an equilibrium state (Table S1). The real-time evolution of the heater temperature (T_measure_) was recorded using a thermocouple, and the results are shown in Figure 3f. As the heater was switched on (at 190 s) with an applied power of 20 W, the heater temperature rose steeply and rapidly reached an equilibrium state. The highest temperature value was 364.8 K without TIM packaging of the system; the use of GP as a TIM markedly reduced the heater temperature to 327.2 K, revealing the important role of the TIM for enhancing the heat dissipation capacity of the cooling system. Compared with GP, DGP demonstrated a superior heat dissipation performance, exhibiting a further temperature drop of 6.2 K (heater temperature: 321.0 K). As shown in Figure 3g, the equivalent heat transfer coefficients for the cooling systems with and without the TIMs were calculated from the linear variation between equilibrium heater temperature and the applied power, which is equal to the reciprocal of the slope. The equivalent heat transfer coefficients for the three cases were 0.3, 0.69, and 0.87 W K^−1^, respectively, indicating that DGP achieved a 26% enhancement of cooling efficiency compared with GP.

To reveal the change in the thermal contact resistance (R_c_) between graphene paper before and after decoration, a commercial computational fluid dynamics software (Icepak) was used for in-depth thermal analysis of our test system at an operating power of 20 W, the detailed parameter settings and simulation model of which are shown in Figure S3. The effective thermal conductivity (*κ_ef_*_f_) of GP and DGP from the simulation results were 2.8 and 4.3 W m^−1^ K^−1^, respectively, as shown in Figure 3h. The thermal contact resistance can be calculated using the equation *R*_c_ = *R_TIM_ − R*_Bulk_ *= BLT*/*κ_eff_* − *BLT*/*κ_bulk_*, where *κ_bulk_* is the intrinsic thermal conductivity of graphene paper under a compression of 500 kPa [46]. The schematic diagram to show the composition of the thermal interface resistance and the calculation results can be found in Figure S4 and Table S2. The thermal conductivity of this graphene paper measured used the laser flash method was 5.8 W m^−1^ K^−1^, which gives a low thermal contact resistance for the DGP of 15 K mm^2^ W^−1^. Under the same conditions, the thermal contact resistance of GP was 46 mm^2^ W^−1^, which is approximately three times higher than that of DGP, indicating that the decoration process significantly decreased the thermal contact resistance between graphene paper and the mating surface. Figure 3i shows the change in the heater temperature with the packaging pressure for GP and DGP. With decreasing packaging pressure, the heater temperature increased, which can be attributed to the increasing thermal contact resistance caused by inadequate contact between the TIM and the mating surface. Noticeably, compared with GP, the heater temperature exhibited fewer changes with DGP (5.8 K:12.6 K at 100 kPa), which can be attributed to the adhesion of PDA that enhances contact between the DGP and the mating surface at low packaging pressure.

**Figure 4:**
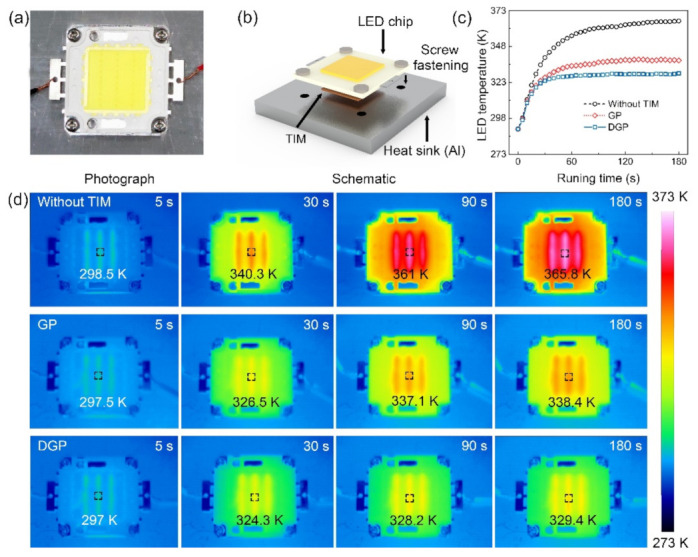
(**a**) Experimental setup and (**b**) schematic configuration for comparing the cooling efficiency between GP and DGP based on the heat dissipation of an LED. (**c**) Temperature evolution of the LED chip as a function of running time, with the corresponding IR images shown in (**d**).

Given the practical application of TIMs, the different heat dissipation capacities of GP and DGP were investigated for a high-power light emitting diode (LED, rated power: 30 W). As shown in Figure 4a,b, GP and DGP with the equal lateral size (2.5 × 2.5 cm^2^) and BLT (250 μm) were physically fastened between the Al heat sink and LED chip using four screws. Heat generated by the operating LED was extracted by the air-cooling system to reach an equilibrium state. Real-time evolution of the LED temperature was recorded using a calibrated infrared (IR) camera, as shown in Figure 4c. It is clear that the temperature increases more slowly when using DGP as the TIM than GP, and it continuously shows a lower value. At the equilibrium state (180 s), when using DGP, the temperature of the LED exhibited a further decrease of 9 K compared with GP (from 338.4 K to 329.4 K), which can be attributed to the decoration that reduces the thermal contact resistance between GP and the mating surface. It should be noted that the reliability of the LED chip depends exponentially on the operating temperature, where a temperature decrease of 10 K can extend the LED lifespan by 50% [47,48]. Thus, the improved heat dissipation indicates that the decoration of the graphene-based TIM with PDA-EGaIn nanocapsules is a promising way to enhance the TIM performance for practical electronic cooling applications.

## 4. Conclusions

A graphene paper fabricated via a facile filtration method was decorated with PDA-EGaIn nanocapsules, which were prepared via sonication of bulk EGaIn in an aqueous dopamine hydrochloride solution. The dopamine served as a surfactant to stabilize the EGaIn nanodroplets during the sonication process and also served to immobilize the EGaIn nanocapsules on the graphene paper surface. The decorated graphene paper was applied as a TIM and demonstrated a 26% enhancement in cooling efficiency compared with graphene paper. The simulation results indicated that this enhancement was because of the lower thermal contact resistance (15 K mm^2^ W^−1^) between DGP and the mating surface compared with GP (46 K mm^2^ W^−1^). The decrease in the thermal contact resistance between the decorated TIM and mating surface was more remarkable at low compression pressures. The clear enhancement in the heat dissipation performance after the TIM is decorated with PDA-EGaIn nanocapsules indicates that this method is promising for improving the heat dissipation capacity of graphene-based TIMs for practical electronic cooling applications.

## Supplementary Materials

The following are available online at https://www.mdpi.com/article/10.3390/nano11051236/s1, Figure S1. The zeta potential of EGaIn nanodroplet (a) without dopamine, (b) before adding Tris and (c) after adding Tris; Figure S2. SEM image of the (a) EGaIn nanocapsules with PDA coating (b) EGaIn nanodroplet without PDA coating; Figure S3. (a,b) Icepak system model of TIM performance evaluation. (c) Top-view and (d) cross-sectional temperature distribution of the simulated system with GP as TIM. (e,f) The case when DGP is used as TIM; Figure S4. A schematic diagram to show the composition of the thermal interface resistance; Table S1. The detailed settings of the heater, TIM and the heat sink; Table S2. The calculated thermal interface resistance (RTIM) and thermal contact resistance (Rcontact) of the three applied TIMs.
