# Supplementary material for: Surface Modification Using Polydopamine-Coated Liquid Metal Nanocapsules for Improving Performance of Graphene Paper-Based Thermal Interface Materials
Source: Nanomaterials (Basel). 2021 May 7;11(5):1236. doi: 10.3390/nano11051236 (PMC8151624; doi:10.3390/nano11051236)
Supplement: Supplementary file 1 [file nanomaterials-11-01236-s001.zip › nanomaterials-1172776-supplementary.pdf]

Supplementary Materials

Surface modification using polydopamine-coated liquid metal nanocapsules for improving performance of graphene paper-based thermal interface materials

Jingyao Gao[a,b,1], Qingwei Yan[a,c,1], Xue Tan[a,b], Le Lv[a,b], Jufeng Ying[a,b], Xiaoxuan Zhang[a,b], Minghui Yang[d], Shiyu Du[e], Qiuping Wei[f], Chen Xue[a,b], He Li[a,b], Jinhong Yu[a,b], Cheng-Te Lin[a,b,*], Wen Dai[a,b,*], Nan Jiang[a,b,*]

[a] *Key Laboratory of Marine Materials and Related Technologies, Zhejiang Key Laboratory of Marine Materials and Protective Technologies, Ningbo Institute of Materials Technology and Engineering, Chinese Academy of Sciences, Ningbo 315201, China.*

[b] *Center of Materials Science and Optoelectronics Engineering, University of Chinese Academy of Sciences, Beijing 100049, P.R. China.*

[c] *College of Materials Science and Engineering, Hunan University, Changsha, 410082, P. R. China.*

[d] *Ningbo Institute of Materials Technology and Engineering, Chinese Academy of Sciences, Ningbo, China.*

[e] *Engineering Laboratory of Advanced Energy Materials, Ningbo Institute of Materials Technology and Engineering, Chinese Academy of Sciences, Ningbo, Zhejiang, 315201, China.*

[f] *School of Materials Science and Engineering, Central South University, Changsha 410083, P.R. China.*

*Corresponding author, E-mail: jiangnan@nimte.ac.cn; linzhengde@nimte.ac.cn; daiwen@nimte.ac.cn.

[1]Jingyao Gao and Qingwei Yan contributed equally to this work.

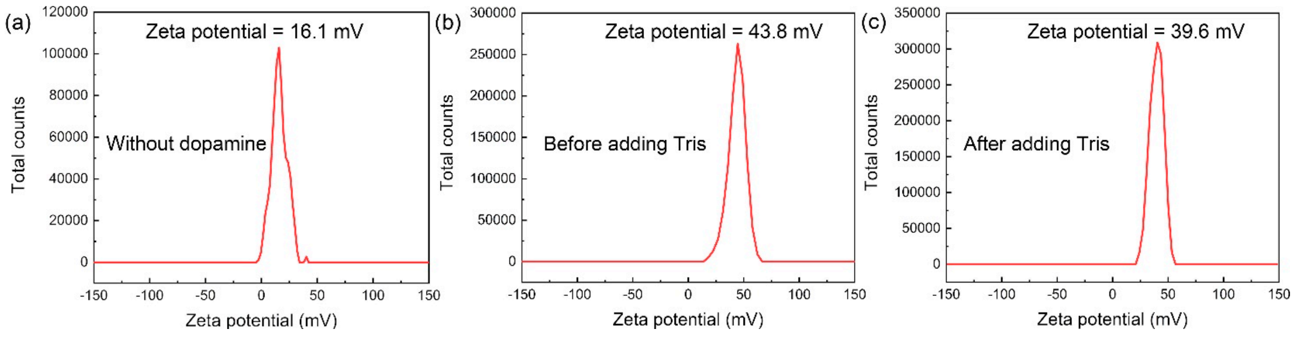

Figure S1. The zeta potential of EGaIn nanodroplet (a) without dopamine, (b) before adding Tris and (c) after adding Tris.

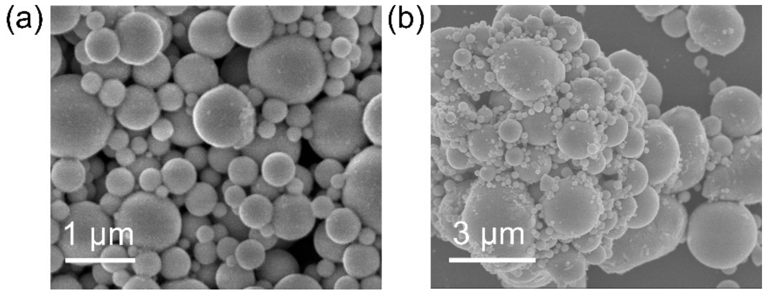

Figure S2. SEM image of the (a) EGaIn nanocapsules with PDA coating (b) EGaIn nanodroplet without PDA coating

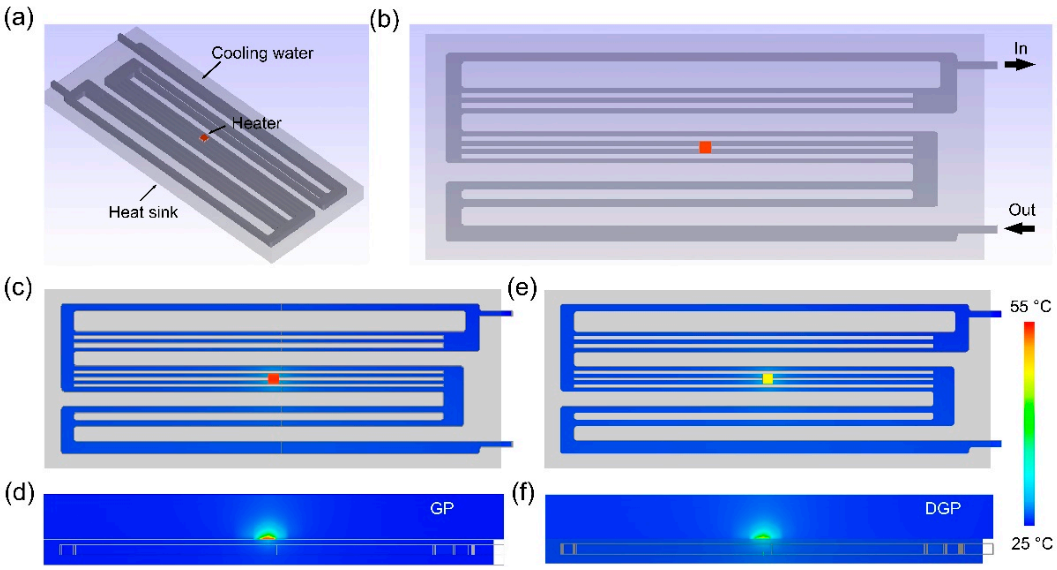

Figure S3. (a and b) Icepak system model of TIM performance evaluation. (c) Top-view and (d) cross-sectional temperature distribution of the simulated system with GP as TIM. (e) and (f) The case when DGP is used as TIM

The heat dissipation capacity of the applied TIMs was further study through a simulation process by using finite element analysis based on a commercial computational fluid dynamics software (Icepak). The model and the corresponding simulated results are shown in **Fig. S3**. The background temperature is 25 °C at 1 atm in atmosphere. The entrance temperature of cooling water is 25 °C. The detailed settings of the heater, TIM and the heat sink are listed as follows:

Table S1. The detailed settings of the heater, TIM and the heat sink

	Size<br>cm^3	Materials	κ<br>$W\ m^{-1}K^{-1}$	C_p<br>$J\ g^{-1}\ K^{-1}$
Heater	1 × 1 × 0.125	Alumina	27	0.91
Heat sink	22.3 × 9 × 1.2	Aluminum alloy	205	0.90
GP	1 × 1 × 0.025	Graphene	2.8	0.75
DGP	1 × 1 × 0.025	Graphene	4.3	0.75

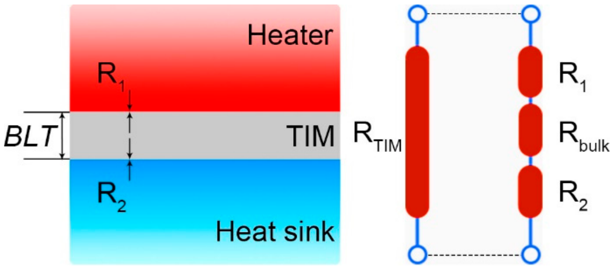

Figure S4. A schematic diagram to show the composition of the thermal interface resistance

The thermal contact resistance ($R_{contact}$) of TIMs is calculated using the Equation S1, with detailed parameters and results shown in **Table S2**. Note that the bond line thickness (*BLT*) means the thickness of the samples when used as TIMs under packaging conditions.

$$R_{contact} = R_1 + R_2 = R_{TIM} \text{-} R_{bulk} = \frac{BLT}{\kappa_{eff}} - \frac{BLT}{\kappa_{bulk}} \qquad (S1)$$

Table S2. The calculated thermal interface resistance (R_{TIM}) and thermal contact resistance ($R_{contact}$) of the three applied TIMs.

	BLT<br>μm	κ_{bulk}<br>$W\ m^{-1}K^{-1}$	R_{bulk}<br>$K\ mm^2\ W^{-1}$	κ_{eff}<br>$W\ m^{-1}K^{-1}$	R_{TIM}<br>$K\ mm^2\ W^{-1}$	$R_{contact}$<br>$K\ mm^2\ W^{-1}$
DGP	250	5.8	43	4.3	58	15
GP	250	5.8	43	2.8	89	46
